# Is methane a new therapeutic gas?

**DOI:** 10.1186/2045-9912-2-25

**Published:** 2012-09-25

**Authors:** Wenwu Liu, Dong Wang, Hengyi Tao, XueJun Sun

**Affiliations:** 1 Department of Diving Medicine, Second Military Medical University, 800 Xiangyin Road, Shanghai 200433, P.R. China; 2 Institute of Nautical Medicine, Nantong University, Jiangsu, 226019, P.R. China; 3 Department of Physiology and Pharmacology, Loma Linda University School of Medicine, Loma Linda, CA 92354, USA

**Keywords:** Methane, Anti-inflammation, Therapeutic gas, Methanogen

## Abstract

**Background:**

Methane is an attractive fuel. Biologically, methanogens in the colon can use carbon dioxide and hydrogen to produce methane as a by-product. It was previously considered that methane is not utilized by humans. However, in a recent study, results demonstrated that methane could exert anti-inflammatory effects in a dog small intestinal ischemia-reperfusion model.

**Point of view:**

Actually, the bioactivity of methane has been investigated in gastrointestinal diseases, but the exact mechanism underlying the anti-inflammatory effects is required to be further elucidated. Methane can cross the membrane and is easy to collect due to its abundance in natural gas. Although methane is flammable, saline rich in methane can be prepared for clinical use. These seem to be good news in application of methane as a therapeutic gas.

**Conclusion:**

Several problems should be resolved before its wide application in clinical practice.

## Introduction

In a recent study of Boros et al., methane was shown to confer protective effect on the oxidative stress and inflammation in ischemic and reperfusion induced intestinal injury [1]. In the methane treated canines, Boros et al. demonstrated that inhalation of 2.5% methane for 15 min significantly ameliorated the histological damage to the intestinal mucosa and dramatically decreased the myeloperoxidase, a marker for oxidative stress and neutrophilic infiltration. In the *in vitro* experiments, results showed incubation with 2.5% methane at normal pressure could inhibit the superoxide production in cultured primary canine granulocytes following stimulation. They also noted that 2.5% methane inhalation for 3 h had no side effects on the blood gas chemistry and no influence on the macrohemodynamics in unstressed rats.

### Characteristics and synthesis of methane

Methane is a chemical compound with the chemical formula CH_4_. It was discovered and isolated by Alessandro Volta between 1776 and 1778 when studying marsh gas from Lake Maggiore. Methane is the simplest alkane, the main component of natural gas (about 87% by volume), and probably the most abundant organic compound on earth [2]. Thus, it has been an attractive fuel. In the chemical industry, methane is converted to synthesis gas, a mixture of carbon monoxide (CO) and hydrogen (H_2_), by steam reforming. Methane is non-toxic; however, it is extremely flammable and may form explosive mixtures with air: it is flammable only over a narrow range of concentrations (5–15%) in air [3].

Methane can be synthesized in biological route and industrial route. In the industrial route, methane is produced by hydrogenating carbon dioxide (CO_2_) through the Sabatier process. However, the intentional production of methane is relatively rare due to its abundance in natural gas. In the biological route, methanogens in the colon can use CO_2_ and H_2_ to produce methane as a by-product [4]. Methane gas is produced by enteric bacteria in 30–62% of humans [5] and some studies have focused on the relationship between methane production and intestinal diseases such as constipation predominant irritable bowel syndrome (C-IBS), diverticulosis and colon cancer [4,6,7]. In addition, Ghyczy et al. found that the rat mitochondrial subfractions and endothelial cell cultures under hypoxic conditions had methane generation, which represented an alternative approach to methanogenesis [8].

Methane is thought to be produced exclusively by anaerobic fermentation in the gut [9]. It was previously considered that methane is not utilized by humans (inert or biologically inactive), so it is excreted either as flatus, or it traverses the intestinal mucosa and is absorbed into the systemic circulation and excreted unchanged through the lungs. In a study, pulmonary methane excretion ranged from undetectable to 0.66 ml/min, and 20% of total methane produced was excreted via the lungs [10]. This is the basis of lactulose breath test (LBT). Due to its ease of administration and minimal invasiveness, breath testing has become a widely used tool in diagnosis of certain gastrointestinal conditions and disorders of transit [6].

### Biology of methane

In past decades, there has been extraordinary, rapid growth in our knowledge on gaseous molecules, including nitric oxide (NO), CO and hydrogen sulfide (H_2_S), which have been known to play important roles in the biological systems [11]. Actually, the bioactivity of methane is not a novel phenomenon in medicine. Studies have shown that methane can slow the intestinal transit by altering intestinal neuromuscular function [5] and decrease peristaltic velocity and increase contraction amplitude significantly of guinea pig ileum [7].

The Occupational Safety Hazards Administration of United States has regarded methane as a simple asphyxiant, which is intrinsically nontoxic. This seems to be good news when thinking about using this gas as a therapeutic agent. In addition, methane has favorable distribution characteristics with its physical ability to penetrate the membranes and diffuse into the organelles [5]. Excessive oxidative stress is a major cause of some diseases in which the mitochondrial respiratory chain is a major source of devastating reactive oxygen species (ROS), but antioxidants have limited therapeutic efficacy which may be attributed to the impermeability of membranes to them [12]. As methane can reach the nucleus and mitochondria, the protection of nuclear DNA and mitochondria suggests the protective effect of methane on the oxidative stress-related diseases.

Furthermore, Pimentel et al. have hypothesized that methane might confer an effect on membrane channels because halothane, a hydrocarbon gas similar to methane, has known demonstrable effects on G-proteins, membrane or intracellular processing of the receptor signal and acetylcholine activated ion channel kinetics [13-15]. In the study of Boros et al. [1], they hypothesized that methane may accumulate transiently at cell membrane interfaces, thereby change the physicochemical properties or the *in situ* functionality of proteins embedded within this environment, which may influence the function of membrane bound enzymes, including xanthine oxidoreductase or those leading to ROS formation. Fink also proposed that whether mammalian cells contain an oxygenase that is capable of using methane as a substrate, whether the biological effects of methane are caused by the formation of small amounts of the reactive alcohol, methanol, and/or changes in the redox milieu of the cell due to changes in NAD(P)^+^/NAD(P)H ratio, and whether there is a cellular “receptor” for methane are required to be elucidated [16]. Thus, studies with elegant design are required to confirm the exact molecular mechanism underlying the protective effect of methane.

### Issues in application of methane as a therapeutic gas

As above mentioned, the specific mechanism of the therapeutic effect of methane is required to be further elucidated in future studies. In addition, the therapeutic effect of methane may be confirmed in other animal models to elucidate whether the protective effect of methane is species-specific.

The methane is abundant in natural gas and its separation (production) is relatively easy. This is different from xenon which is scarce in the atmosphere significantly limiting the wide application of xenon as an inhaled anesthetic. These advantageous characteristics make methane very appealing as an inhaled gas for therapeutic purposes. Since inhaled methane acts more rapidly, methane inhalation may be suitable for defense against acute oxidative stress. However, methane inhalation may be impractical for daily application for disease prevention due to its flammability and difficulty to transport. Thus, normal saline rich in methane may be prepared as an injection which may be portable, easily administered and safe. This has been realized in application of H_2_[17].

Of note, not all administered methane is degraded *in vivo* and a majority of methane will be eliminated via the lungs or the skin [18]. However, recent attention has been paid to the potential of methane to contribute to climatic change and global warming. Atmospheric methane concentrations were stable until about 100 years ago when concentrations began to rise. In 1992, it was estimated methane would cause 15–17% of global warming over the next 50 years [19] and methane has a global warming potential of 25 compared to CO_2_ over a 100-year period (although accepted figures probably represents an underestimate [20]. Thus, the recycle of methane should be considered once it has been used as a therapeutic gas.

In addition, whether administration of exogenous methane may affect the production and excretion of endogenous methane is another concern for methane treatment. In the gastrointestinal diseases, methane has been regarded as a “bad guy” [4] and whether administration of methane increases the risk for these diseases is unclear. If the administration of exogenous methane affects the production of methane in the colon, the constituents of flatus might be changed and whether this may cause imbalance among constituents of flatus is still unclear. We assume that methane treatment for a long time or with a high frequency might be detrimental, and the influence of methane treatment for a short time on intestinal flora or risk for gastrointestinal diseases is needed to be further investigated.
